# Modern agriculture and One Health

**DOI:** 10.1186/s40249-024-01240-1

**Published:** 2024-10-10

**Authors:** Guangzhi Zhang, Yu Qiu, Pascal Boireau, Yinghui Zhang, Xin Ma, Hui Jiang, Ting Xin, Maodun Zhang, Zelalem Tadesse, Nisar Ahmad Wani, Junxia Song, Jiabo Ding

**Affiliations:** 1 Institute of Animal Sciences, Chinese Academy of Agricultural Sciences, Beijing, 100193 China; 2 Animal Production and Health Division, Food and Agriculture Organization of the United Nations, 00153 Rome, Italy; 3 ANSES, Laboratoire de Santé Animale, 94700 Maisons-Alfort, France; 4 Institute of Veterinary Drug Control, Beijing, 100193 China; 5 University of California, Berkeley, CA 94720-3200 USA; 6 Reproductive Biotechnology Center, Dubai, 299003 UAE

**Keywords:** Modern agriculture, One Health, Ecosystem, Interspecies disease transmission, Antimicrobial resistance, Sustainable agrifood systems transformation

## Abstract

**Background:**

The development of modern agriculture has significantly contributed to improving global food security and safety, alleviating poverty, and enhancing human health and livelihoods. However, the rapid advancement of modern agriculture has also brought about various challenges that limit its sustainable development. This commentary aims to discuss these issues through the One Health lens, and provide valuable insights for balancing modern agricultural activities with the need to protect and promote the health of all the sectors.

**Main text:**

This commentary explores the multifaceted impacts of modern agriculture on social development, as well as the associated various health challenges and environmental impacts within the One Health framework. Key issues include ecosystem degradation, increased risk of interspecies disease transmission like zoonoses, reverse zoonoses, and vector-borne diseases, and the escalated threat of antimicrobial resistance due to intensified agricultural production and increased antimicrobial use. To address these challenges, this commentary outlines potential solutions anchored in the development and implementation of modern technologies and good agricultural practices, such as precision farming, integrated pest management, biosecurity measures, vaccination programs, as well as surveillance and early detection of health risks.

**Conclusions:**

Good agricultural practices supported by scientific and technological advancements are essential for aligning productivity with the One Health vision, ensuring the health and resilience of all the sectors. Enhancing stakeholder education, strengthening regulatory frameworks, and providing supportive policies and infrastructure for farmers to adopt sustainable practices are crucial for the long-term viability of agrifood systems. The Food and Agriculture Organization of the United Nations plays a pivotal role in guiding this sustainable transformation through the One Health approach.

## Background

The development of modern agriculture has been a cornerstone in the progress of human civilization, which plays a pivotal role in improving global food security and safety, alleviating poverty, and enhancing human health and livelihoods. However, the rapid advancement of modern agriculture has also brought about various health challenges and environmental impacts that hinder its sustainable development. In the meantime, the concept of One Health has emerged as a multifaceted approach, advocating for collaborative efforts across disciplines and sectors to optimize the health across human, animal and environmental sectors. This commentary aims to delve into these issues through the One Health perspective, and to provide valuable insights for sustainable agrifood systems transformation that balances agricultural activities with the need to protect and promote the health of all the sectors.

## Main text

### Modern agriculture supports social development

Agriculture is one of the primary income resources in many countries, and is the main driver of development in most rural areas. Despite the rapid growth of the global population, which has surged from less than three billion people in 1950 to over eight billion today, food security levels worldwide have managed to remain stable. This remarkable achievement is largely attributed to the development and wide adoption of modern agricultural technologies. Modern agriculture operates at the forefront of scientific progress, which includes mechanization, biological and digital technologies, value chain development, intensified production methods, tailored nutrition programs, comprehensive health services, pharmaceutical interventions, chemical fertilizers, etc. By leveraging these cutting-edge technologies, modern agriculture enhances productivity, efficiency, and profitability per unit of resource and labor input.

Modern agriculture also includes stringent food safety management measures to ensure that food is safe for consumption. For example, the development of cold chain and infrastructural advancement for storage and transportation increase the shelf life of food, prevent contamination, reduce post-harvest losses, and ensure products reach markets in optimal conditions. Slaughterhouse inspection also plays a critical role in guaranteeing meat safety before it enters the food chain. Notably, modern agricultural system not only ensures a steady and abundant supply of nutritious food, but also facilitates its equitable distribution. Moreover, specialization and value chain development in modern agriculture promote professional education and training, support rural community development, improve household income and livelihoods, and strengthen overall well-being and social resilience. As such, modern agriculture plays a pivotal role in reducing poverty, improving economic equity, and achieving the United Nations Sustainable Development Goals (SDGs).

### Addressing ecohealth challenges in modern agriculture

While the world is benefiting significantly from modern agricultural technologies, the agricultural development is also associated with various One Health challenges including the ecosystem degradation. Practices such as intensive farming, monocropping, and intensive use of agrochemicals like chemical fertilizers and pesticides can lead to soil erosion, water pollution, and loss of biodiversity. Environmental exposure of humans to excessive agrichemicals can result in both acute and chronic health effects, such as gastrointestinal diseases, allergies, birth defects, cancer, neurological disorders, etc [1]. Additionally, the agrifood systems account for one-third of total anthropogenic greenhouse gas emissions [2], posing a significant impact on climate change. These activities not only undermine the health of ecosystems but also threaten the long-term sustainability of food production. As an example, increased temperature can reduce yields of desirable crops while encouraging weed and pest proliferation.

To ensure sustainability, it is crucial to promote safe and sustainable agricultural production practices, which are bolstered by scientific and technological advancements. Examples include integrated pest management (IPM) techniques that combine biological, cultural, and mechanical control methods to manage pests in an environmentally friendly way, decreasing reliance on pesticides. Precision farming technologies, such as Global Positioning System-guided equipment and drones, enable more efficient application of water, fertilizers, and pesticides, thus reducing waste and runoff [3]. Modern farm waste management techniques, such as anaerobic digesters and composting systems, can efficiently convert waste into valuable resources while preventing the release of health hazards into the environment [4].

### Addressing challenges of interspecies disease transmission in modern agriculture

Modern agricultural development, encompassing habitat destruction, land-use changes, intensive farming, and increased travel and trade, can significantly increase opportunities for close interactions between humans, livestock, and wildlife. These interactions, combined with the ecological disturbances caused by agricultural activities, accelerate the emergence and interspecies spread of pathogens, posing substantial health risks to both human and animal populations. Indeed, the majority of emerging infectious diseases in humans originate from animals, especially wildlife [5]. Examples include Nipah virus, severe acute respiratory syndrome coronavirus (SARS-CoV), Middle East respiratory syndrome virus, monkeypox virus, highly pathogenic avian influenza virus H5N1, etc [6]. Similarly, the risk for reverse zoonotic transmission of pathogens from humans to other vertebrates also increases [7]. Examples include the transmission of *Mycobacterium tuberculosis*, Methicillin-resistant *Staphylococcus aureus*, 2009 pandemic H1N1 influenza virus, SARS-CoV-2, *Cryptosporidium parvum,* or *Giardia duodenalis* from humans to animals [7, 8]. Irrigated agriculture also poses public health risks associated with vector-borne diseases. Paddy fields, irrigation systems, and peridomestic environment facilitate the breeding of mosquitoes for transmitting diseases such as Rift Valley fever (RVF), West Nile disease, malaria, Japanese encephalitis, and dengue [9]. In some circumstances, livestock hosts serve as amplifiers for vector-borne pathogens, such as pigs for Japanese encephalitis and ruminants for RVF, increasing the infection risk to humans living nearby.

Although modern agricultural development is associated with an elevated risk of the emergence and interspecies spread of diseases, it also offers solutions through innovations in technology and scientific progress. For example, the development and widespread use of vaccines and biosecurity measures significantly contribute to mitigated disease risk and improved animal health and production. By implementing mass vaccination with good quality vaccines, many countries have successfully eliminated some high-impact diseases in domestic animals such as rinderpest, foot and mouth disease, brucellosis, lumpy skin disease, rabies, etc. By improving biosecurity, such as controlling access to farms, pest and vermin control, cleaning and disinfection, farms can significantly reduce the chance of pathogen introduction and spread. Additionally, modern agricultural system includes routine veterinary service and health monitoring. Implementation of robust surveillance for zoonotic diseases in domestic animal hosts enables timely control measures and prevents spillover transmission to humans [10]. Furthermore, modern agricultural system emphasizes food safety standards and traceability, ensuring that food products are processed and handled hygienically to reduce contamination risks. For example, pasteurization involves heating milk to a specific temperature to kill pathogens like *Mycobacterium bovis*, *Brucella* spp., and *Listeria* spp., thereby preventing illness caused by milk-borne zoonoses. By integrating these methods, modern agriculture improves productivity while safeguarding public health by reducing the risk of zoonotic disease transmission.

### Tackling antimicrobial resistance in modern agriculture

Similar to ecosystem degradation and interspecies transmission of diseases, antimicrobial resistance (AMR) also presents a significant risk to the sustainability of agrifood production and the health of all the sectors. Although AMR occurs naturally, misuse and overuse of antimicrobials in humans, animals, and agriculture can greatly accelerate the development and selection of drug-resistant microorganisms. This exposes humans, animals, and crops to the risk of diminished effectiveness of antimicrobials as therapeutic agents. Antimicrobial use (AMU) was considered a hallmark of modern animal husbandry, but it has become highly controversial due to the increasing attention over AMR and One Health. Besides disease treatment, antimicrobials are used in animals for disease prevention and control, usually at sub-therapeutical levels. In some countries, antimicrobials are still used as feed additives to improve feed conversion efficiency and promote animal growth [11]. Antimicrobial use in food-producing animals tends to be higher in intensified production systems with suboptimal biosecurity measures compared to low-input and low-output production systems [12]. Several factors contribute to this disparity. Intensified livestock and aquaculture production often involves homogenized genetic breeds, crowded and stressed conditions, compromised animal immune status, and increased susceptibility to infections. Hence, antimicrobials are often systematically and routinely applied to compensate for inadequate animal husbandry practices and poor hygiene. Additionally, economic pressure and the drive to maintain high production levels lead to using antimicrobials as an easy and cheap approach to prevent potential losses from diseases and stimulate growth, which are often prioritized over AMR risk mitigation. In contrast, smallholder extensive farms often employ a diversified and less dense farming approach, leading to less susceptible animals and, hence, reduced need for routine antimicrobial administration. Despite the relatively higher demand for antimicrobials, modern and intensified production systems have distinct advantages in detecting AMR and managing associated risks. These farms typically have access to disease diagnostic facilities, drug susceptibility testing, and specialized veterinary services for disease prevention, health monitoring, as well as guidance on AMU when treatment is necessary. In contrast, traditional subsistence farms often lack such support and tend to rely on self-medication and peer learning, increasing the risk of AMR emergence [13].

Antimicrobial resistance can spread across the human-animal-plant-environmental interface, necessaring the integrated, cross-sectoral One Health approach to tackle the risk. The quantified contribution of AMU and AMR in agricultural production to the AMR risk in humans remains to be identified, although livestock workers tend to have higher levels of AMR compared to people not regularly exposed to livestock [14]. In addition, farm wastes and animal manure contain antimicrobial residues, resistant microorganisms, and resistance genes, which can contaminate the environment if they are left untreated to fertilize cropland or released into waterways. Furthermore, antimicrobials administered to aquaculture and plants are directly released into the environment, and changes in the surrounding microbial biodiversity have been noted [15]. All those can contribute to the development of environmental reservoirs of AMR and vectors for its spread.

To tackle AMR in agrifood systems effectively, it is essential to adopt comprehensive and integrated strategies following the One Health approach. This includes adopting good agricultural practices to reduce the need for antimicrobials, such as improving animal husbandry and health services, enhancing biosecurity measures and disease early detection, and promoting the use of quality vaccines. Additionally, regulations and surveillance programs to guide, control and monitor the use of antimicrobials are also needed to prevent misuse and overuse in agriculture. Educating key stakeholders, including farmers and health providers, about the risk of AMR and promoting alternatives to antimicrobials can further support this effort. As a guiding strategy to tackle AMR in countries, the National Action Plan on AMR shall emphasize the multi-sectoral approach, and strengthen the inclusiveness and contribution of agrifood and environmental sectors in the joint battle [16]. Integration of AMR into countries’ wider sustainable agrifood systems transformation would help ensure long-term achievements and impacts.

## Conclusions

Agriculture is fundamental to social development, human health, and overall well-being. To be sustainable, agriculture must meet the needs of present and future generations, while ensuring profitability and balanced health across all the sectors. To manage the challenges associated with modern agricultural development, good agricultural practices should be promoted, such as precision farming, proper waste management, prudent use of antimicrobials and pesticides, biosecurity, vaccination, health monitoring and surveillance, etc. The One Health approach, which recognizes the interconnectedness of human, animal, and environmental health, can effectively align with modern agricultural development to support its sustainability. Advancements in science and technology catalyze this process, leading to improved productivity and mitigated negative health and environmental impacts. Meanwhile, training and education programs for farmers, veterinarians, and agricultural stakeholders are needed to improve their awareness of One Health and the adoption of sustainable practices. Proper regulation, effective governance, and supportive policies and infrastructure are also crucial to ensure compliance with safety standards, animal welfare, and environmental protection.

As a global leading organization in defeating hunger and achieving food security for all, the Food and Agriculture Organization of the United Nations (FAO) promotes evidence-based, country-owned One Health interventions to accelerate sustainable agrifood systems transformation and a more resilient and balanced future. Among all, FAO has launched a 10-year global initiative "Reducing the Need for Antimicrobials on Farms for Sustainable Agrifood Systems Transformation (RENOFARM)" in 2024. This global initiative aims to encourage farm-centered approaches to catalyze healthier and more sustainable production, with reduced disease risk and reliance on antimicrobials across all agrifood sectors [17]. Additionally, FAO collaborates closely with partners, including the World Health Organization and other Implementing Entities, through the Pandemic Fund initiative. This collaboration aims to strengthen countries’ animal health systems and capacities to effectively prevent, prepare for, and respond to future pandemic threats at the animal sources, including zoonotic diseases and AMR [18].

## Data Availability

Not applicable.

## Abbreviations

SDGs: Sustainable development goals
IPM: Integrated pest management
SARS-CoV: Severe acute respiratory syndrome coronavirus
RVF: Rift Valley fever
AMR: Antimicrobial resistance
AMU: Antimicrobial use
FAO: Food and Agriculture Organization of the United Nations
RENOFARM: Reducing the Need for Antimicrobials on Farms for Sustainable Agrifood Systems Transformation
